# Bovine milk with variant β-casein types on immunological mediated intestinal changes and gut health of mice

**DOI:** 10.3389/fnut.2022.970685

**Published:** 2022-09-30

**Authors:** Bin Liu, Weicang Qiao, Minghui Zhang, Yanpin Liu, Junying Zhao, Lijun Chen

**Affiliations:** ^1^National Engineering Research Center of Dairy Health for Maternal and Child, Beijing Sanyuan Foods Co., Ltd., Beijing, China; ^2^Beijing Engineering Research Center of Dairy, Beijing Technical Innovation Center of Human Milk Research, Beijing Sanyuan Foods Co., Ltd., Beijing, China

**Keywords:** β-casein, morphology, gut microbiome, SCFAs, immune factors, inflammation

## Abstract

Dietary proteins provide bioactive peptides, which are important for host gastrointestinal functions. We hypothesized that A2-type β-casein could provide gastrointestinal benefits and improve the immune and gut health. This study was conducted to investigate those effects and mechanisms. Thirty BALB-c mice (3–4 weeks old) were fed with either a control diet (control), a diet supplemented with bovine milk containing A1 and A2 type β-casein (A1A2, contains 63.62% A2 β-casein of total β-casein) or a diet containing A2 type β-casein (A2A2, contains 95.96% A2 β-casein of total β-casein) (10 ml/kg body weight) for 4 weeks. Immunoglobulin and inflammation factors were measured in serum, and histological variations were measured in duodenal and ileum, and stool 16S rRNA and short-chain fatty acids (SCFAs) contents were measured in fecal samples. Results showed that consumption of A2-type β-casein milk could improve proximal small intestine villus and crypt morphology (*p* < 0.05), increase IgG and IgE responses, and modulate the composition and diversity of gut microbiota by increase the relative abundance of phylum Proteobacteria, class *Clostridia*, family *Ruminococcaceae* and species *Lactobacillus animalis* (*p* < 0.05). There were also significant associations between gut microbes, immune response, and SCFAs, especially isobutyric acid (*p* < 0.05), which may potentially regulated gastrointestinal benefits. Moreover, intake of A2-type β-casein milk had no impact on inflammation. These findings explained potential benefits of consumption of A2-type β-casein milk on host immune system and gut health outcomes, and provide insights to the future application of nutritional modulation.

## Introduction

Milk is rich in high quality protein, calcium, and other beneficial bioactive components, which could potentially promote health condition for humans. Generally, cow’s milk contains ∼3.0 g of protein per 100 g of milk, of which 80% is casein and 20% is whey protein (1). Of the casein in milk, 30–45% is β-casein. Among 12 genetic variants (including A1, A2, A3, B, C, D, E, F, H1, H2, I, and G) identified in bovine milk, the most common types are A1 and A2 β-casein (2, 3). The A1 type differs from the A2 type by a proline-to-histidine amino acid mutation at position 67, which producing a β-casomorphin peptide BCM-7 after gastrointestinal digestion of β-casein (4, 5). The gut releases little or minimal BCM-7 upon intake of A2 genetic variants type milk or milk products. BCM-7 was found to be related with a range of gastrointestinal disorders, such as abdominal pain and altered stool consistency (6–9), and other diseases such as type-1 diabetes (10), and may also cause functional disorders of immune response, digestion and respiration (7, 11).

Several studies in animal models explored the effects of A1 and A2 β-casein on gastrointestinal activities. Barnett et al. showed that A1 β-casein consumption has direct effects on gastrointestinal function via opioid-dependent (gastrointestinal transit and myeloperoxidase activity) and opioid-independent (DPP-4 activity) pathways (6). Milk with exclusively A2 β-casein showed significant positive effects on gut immunology and morphology of an aging mice model (12). Compared with A2 form, A1 form of cow milk has a proinflammatory effect on the lung resulting in phenotype closely matching with the typical allergic asthma phenotype (13). Dairy milk containing A1 variant of β-casein milk could induce inflammation of mice airway respiratory tract, while A1A2 milk had intermediate effects and A2A2 milk induced no inflammation but rather seemed to have a protective effect (13). Consumption of A1 “like” variants of β-casein induced inflammatory response in gut by activating Th2 pathway as compared to A2A2 variants (7).

The aim of this study was to explore the effect of conventional milk, containing the A1 and A2 β-casein, compared to milk containing only the A2 β-casein in an animal model, to explore whether consumption of different β-casein types bovine milk could impact immune and inflammation factors, intestinal morphology and gut short-chain fatty acids (SCFAs) and microbiome.

## Materials and methods

### Milk β-casein and nutritional composition

Cow with its milk containing only A2 β-casein were genetically selected in Beijing Dairy Cow Center, China. Milk samples with both types of β-casein were processed and provided by Beijing Sanyuan Foods Co., Ltd., China. Using isotope labeled peptide and specific standards, β-casein and A2 type β-casein of milk samples were qualified and quantitated using high performance liquid chromatography and Q-Exactive mass spectrometry (Thermo Fisher Scientific, Waltham, MA, USA) (unreleased China Industrial Standard “Analysis of β-casein in Milk and Dairy Products,” #2018-1573T-QB).

Macro-nutrients and micro-nutrients of A1A2 milk and A2A2 milk samples (*n* = 10 for each milk types) were analyzed by using methods according to National Standards of Food in China (Supplementary File 1). Include macronutrients such as protein (Kjeldahl nitrogen method, GB 5009.5-2016, 1st version), fat (Soxhlet extractor method, GB 5009.6-2016, 3rd version), and lactose (HPLC-RID, GB 5009.8-2016, 1st version), and micronutrients such as amino acids (Alanine, Arginine, Aspartate, Cysteine, Glutamate, Glysine, Histine, Isoleucine, Leucine, Lysine, Methionine, Phenylaine alanine, Proline, Serine, Sucine, Tryptophan, Tyrosine, and sum of 18 amino acids, Amino Acid Analyzer, GB 5009.124-2016), and minerals (Calcium, Potassium, Magnesium, Natrium, Phosphorus, Zinc, ICP-MS, GB 5009.268-2016, 2nd version).

### Animals and experimental design

Specific pathogen free (SPF) Balb/c mice (3-week-old, male, *n* = 30, body weight 11–12 g), were housed in 6 cages, and were randomly assigned into 3 experimental groups (10 mice per group), named control, A1A2 and A2A2 groups, receiving standard chow feeding (without milk), standard chow + A1A2 milk feeding, standard chow + A2A2 milk feeding, separately, for 4 weeks. Milk was feed at a dose of 10 ml/kg body weight by oral gavage (5 days/week) according to the previously published A2-beta-casein milk Balb/c mice study (13). The mices were allowed *ad libitum* access to water throughout the experimental period. Health status was monitored daily and body weight was recorded weekly throughout the experiment; feces were collected after the last milk gavage of the experiment, then stored at –80°C for SCFAs analysis and microbiome analysis. At the end of the experiment, mice were fasted overnight and anesthetized by intraperitoneal injection of pentobarbital (10 mg/kg), and blood was collected and centrifuged at 3,000 rpm for 10 min and stored at –80°C environment until further analysis. Protocols were approved by the Animal Care and Use Committee of Sino Animal (Beijing) Science and Technology Development Co., Ltd. (20210077YZE-3R, Beijing, China). Experiment were performed following the Guide of Experimental Animals of the Ministry of Science and Technology (Beijing, China).

### Determination of cytokine and immunoglobulin levels

Enzyme Linked Immunoassorbent Assay (ELISA) was performed according to the manufacturer’s instructions to detect serum levels of cytokines IL-4, IL-5, and IFN-γ, as well as IgG and IgE levels using mice IL-4, IL-5, IFN-γ, IgG, and IgE ELISA kits (Beijing North Institute of Biotechnology Co., Ltd., Beijing, China).

### Histological analysis

Small portions of the middle duodenum and ileum were washed and immediately placed in 10% phosphate-buffered formalin solution and stored at room temperature for histological analysis. The morphological indicators evaluated were: villi height (from the tip of the villi to the crypt), and crypt depth (from the base of the villi to the submucosa). Morphologic analysis was performed on 10 well-oriented and intact villi and 10 crypt foci from each section of duodenum and ileum.

### Short-chain fatty acids analysis

Seven fecal SCFAs (acetic acid, butyric acid, caproic acid, propionic acid, isovaleric acid, and isobutyric acid, and valeric acid) content were quantified using GC/MS as previously described (14).

### Microbial 16S rRNA gene sequencing of the gut microbiome

Bacterial genomic DNA was extracted from 100 mg of homogenized fecal samples using the QIAamp Fast DNA Stool Mini Kit (Qiagen, GmbH, Hilden, Germany) following the manufacturer’s protocol. PCR amplification of the V3-V4 region of 16S rRNA genes (primers 341F: CCTACGGGNGGCWGCAG and 805R: GACTACHVGGGTATCTAATCC) was performed with 10 ng DNA as a template, using 15 μL of Phusion High-Fidelity PCR Master Mix (New England Biolabs), 0.2 μM of forward and reverse primers in a 30 μL total reaction volume. The PCR program included 3 min at 95°C, 25 cycles of 30 s at 95°C and 30 s at 55°C, and then 30 s at 72°C. Sequencing libraries were generated using NEB Next Ultra DNA Library Prep Kit for Illumina (NEB, USA) following manufacturer’s recommendations. Amplicons were sequenced in 2 × 250 bp paired-end reads by Illumina HiSeq 2500 according to the manufacturer’s instructions.

For gut microbiome data, USEARCH (version 1.9) (15) and VSEARCH (version 2.6) (16) were used to quality filter, cluster, and remove chimeras from demultiplexed raw sequencing data. The clustered sequences at 97% similarity level were utilized to construct operational taxonomic units (OTU) tables and representative sequences were assigned taxonomy from phylum to species based on the RDP 16S rRNA training set (Version 16) (17).

### Statistical analysis

Data were analyzed by R language (Verion 3.5.3) (18). Statistical differences of body weights, immune factors, morphological and SCFAs between three groups were performed using Kruskal-Wallis tests followed by Dunn’s *post-hoc* tests. For gut microbiome, data were filtered by removing samples with read numbers less than 1,000 and disregarding taxa from samples with relative abundance less than 0.01% of the sample population. Count data were then normalized using total sum normalization (TSS) by dividing feature read counts by the total number of reads in each sample and transformed by square root transformation (Hellinger transformation). Relative abundance of gut microbial genera, α-diversity, β-diversity, LefSe differential analysis and correlation analysis were performed in R library “microeco” (version 0.5.1) (19). All statistical analysis were considered as significant at *P*-value less than 0.05 level.

## Results

### β-casein and nutritional composition

In both A1A2 and A2A2 milk, the content of A2 β-casein and total β-casein, and their ratio were shown in Supplementary File 1. There were 63.62% A2 β-casein of total β-casein in A1A2 milk, compared with 95.96% of total β-casein in A2A2 milk. Both milk products were similar in nutritional composition, as no significant content differences (*p* < 0.05) of macro- and micro-nutrients were found between A1A2 and A2A2 milk (Supplementary File 1).

### Body weight

**FIGURE 1:**
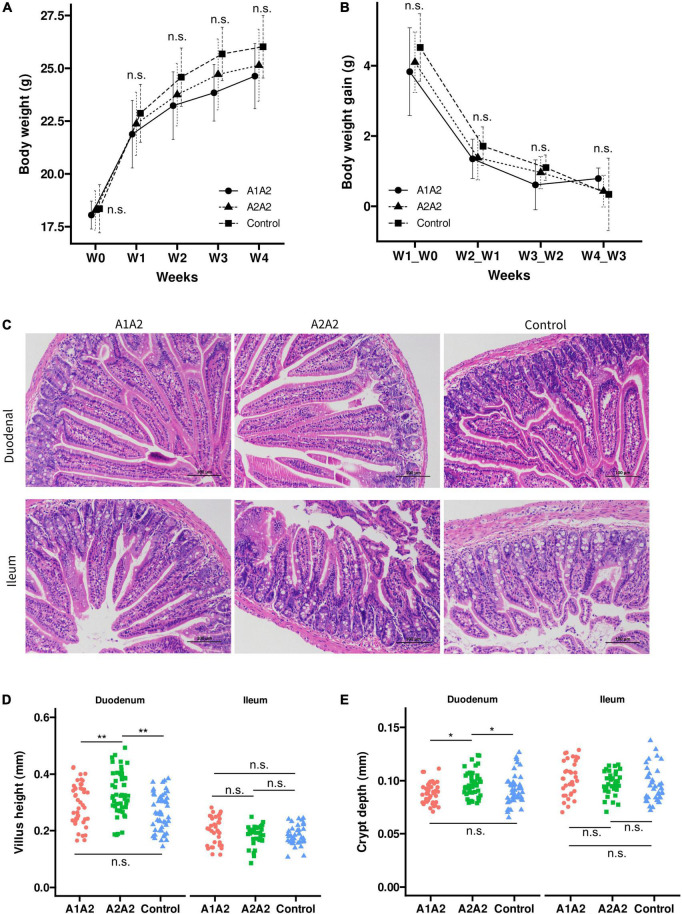
Effects of different types of β-casein milk administration on growth performance **(A)**, weekly body weight gain **(B)**, intestinal morphological structure diagram **(C)**, intestinal villus height **(D)** and crypt depth **(E)** of mice. Control, standard chow feeding (without milk); A1A2, standard chow + bovine milk containing A1 and A2 β-casein; A2A2, standard chow + bovine milk containing only A2 β-casein. Light microscopy (100×) of the intestinal morphology of duodenum and ileum in mice, No histopathological abnormal found in duodenum and ileum of mice in all experimental groups. Kruskal-Wallis tests and Dunn’s *post-hoc* test were performed (*p* at 0.05 level). **p < 0.01, *p < 0.05, n.s., not significant.

To investigate the effects of milk with different β-casein types on the growth performance of mice, we monitored the dynamic growth of mice during experimental period. Compared to the mice in the Control group, mice with β-casein milk administration had no significant difference of body weight on 0, 7, 14, and 21 day (Figure 1A). Moreover, compared with Control group, no significant difference of average daily gains of mice between 0 and 7 day, 7 and 14 day, and 14 and 21 day was found among A1A2 and A2A2 (Figure 1B).

### Morphological analysis

The influence of milk with different β-casein types on the morphometric indices of duodenum and ileum was measured (Figures 1C–E). In duodenum, both villus height and crypt depth were significantly higher in A2A2 group compared with A1A2 and control group. However, in ileum, both villus height and crypt depth showed no significant difference among three groups.

### Serum immune factors and cytokine factors

The concentration of serum immune factors, such as IgG and IgE, and inflammatory cytokine factors, such as IFN-γ, IL-4, and IL-5 were measured. In terms of immunoglobulin, the concentration of IgG and IgE in the A2A2 group were significantly higher than them in the Control and A1A2 groups (*p* < 0.05). However, the concentration of IFN-γ, IL-4, and IL-5 showed no significant difference among three groups after 4 weeks of intervention (Table 1).

**TABLE 1:** Effects of different types of β-casein milk administration on serum immune responses of mice.

Items	A1A2 (*n* = 10)	A2A2 (*n* = 10)	Control (*n* = 10)	*χ* ^2^	*P*	ε^2^
IL-4 (pg/ml)	17.1 ± 6.74	18.62 ± 5.58	17.62 ± 7.8	0.13	0.94	0.004
IL-5 (pg/ml)	13.03 ± 5.57	15.67 ± 5.42	14.75 ± 9.53	0.79	0.67	0.03
IFNγ (pg/ml)	33.79 ± 16.47	39.77 ± 16.08	46.28 ± 19.67	2.59	0.27	0.09
IgG (mg/ml)	5.32 ± 1.3 b	7.18 ± 1.83 a	5.56 ± 1.69 b	7.44	0.024	0.26
IgE (ng/ml)	64.61 ± 17.72 b	82.14 ± 23.89 a	56.04 ± 20.85 b	6.99	0.030	0.24

IgG, immunoglobulin G; IgE, immunoglobulin E; IL-4, interleukin 4; IL-5, interleukin-5; IFN*γ*, interferon *γ*. Control, conventional milk; A1A2, bovine milk containing A1/A2 β-casein; A2A2, bovine milk containing only A2 β-casein. Kruskal-Wallis tests and Dunn’s *post-hoc* test were performed (*p* at 0.05 level). Values labeled with different letters indicate significant difference (*p* < 0.05).

### Fecal short-chain fatty acids

To investigate the gut microbiota metabolism, the fecal SCFAs contents of mice were tested. No significant difference was found in seven fecal SCFAs (acetic acid, butyric acid, caproic acid, propionic acid, isovaleric acid, and isobutyric acid) content among three groups (Table 2).

**TABLE 2:** Effects of different types of β-casein milk administration on fecal SCFAs of mice.

Items (μ g/g)	A1A2 (*n* = 10)	A2A2 (*n* = 10)	Control (*n* = 10)	*χ* ^2^	*P*	ε^2^
Acetic acid	1189.34 ± 123.32	1157.32 ± 197.45	1285.09 ± 197.83	1.98	0.37	0.07
Propionic acid	177.29 ± 43.86	174.8 ± 45.94	179.74 ± 47.4	0.0026	0.99	8.90e-5
Isobutyric acid	16.53 ± 4.16	18.48 ± 5.52	21.08 ± 8.13	2.49	0.29	0.09
Butyric acid	117.35 ± 53.72	117.95 ± 43.45	138.41 ± 98.77	0.16	0.92	0.006
Isovaleric acid	15.29 ± 3.79	16.47 ± 5.34	17.72 ± 5.95	0.61	0.74	0.02
Valeric acid	24.2 ± 9.85	22.83 ± 9.19	28.78 ± 13.23	1.24	0.54	0.04
Caproic acid	1.52 ± 0.29	1.62 ± 0.21	1.65 ± 0.32	1.41	0.49	0.05

Control, conventional milk; A1A2, bovine milk containing A1/A2 β-casein; A2A2, bovine milk containing only A2 β-casein. Kruskal-Wallis tests and Dunn’s *post-hoc* test were performed (*p* at 0.05 level). Values labeled with different letters indicate significant difference (*p* < 0.05).

### Fecal microbiome

The effects of different types of β-casein milk on bacterial compositions of mice were carried out using 16S rRNA sequencing (V3-V4 region). A total of 2,159,398 sequences were obtained from 30 samples. 5176 OTUs were identified at 97% sequence similarity, and then they were assigned to 19 phyla, 37 classes, 63 orders, 113 families, and 218 genera.

**FIGURE 2:**
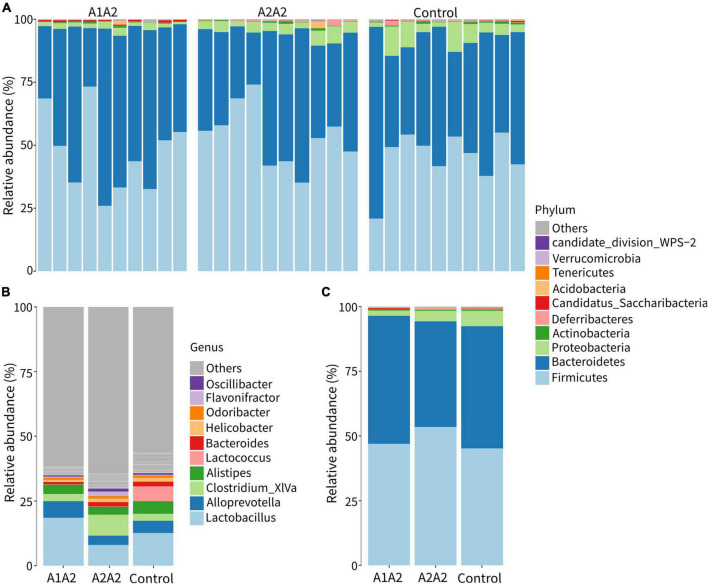
Relative abundances of gut microbial composition at the phylum **(A,C)** and genus level **(B)** of mice on different types of β-casein milk administration. Control, standard chow feeding (without milk); A1A2, standard chow + bovine milk containing A1 and A2 β-casein; A2A2, standard chow + bovine milk containing only A2 β-casein.

Composition analysis showed that Firmicutes (50%), Bacteroidetes (45%), Proteobacteria (<5%) and Actinobacteria (<5%) were the dominant phyla in all samples (Figures 2A,C). At genus level, *Lactobacillus*, *Alloprevotella*, *Clostridium* XIVa, *Alistipes*, *Lactococcus*, and *Bacteroides* were the dominant genera (Figure 2B).

**FIGURE 3:**
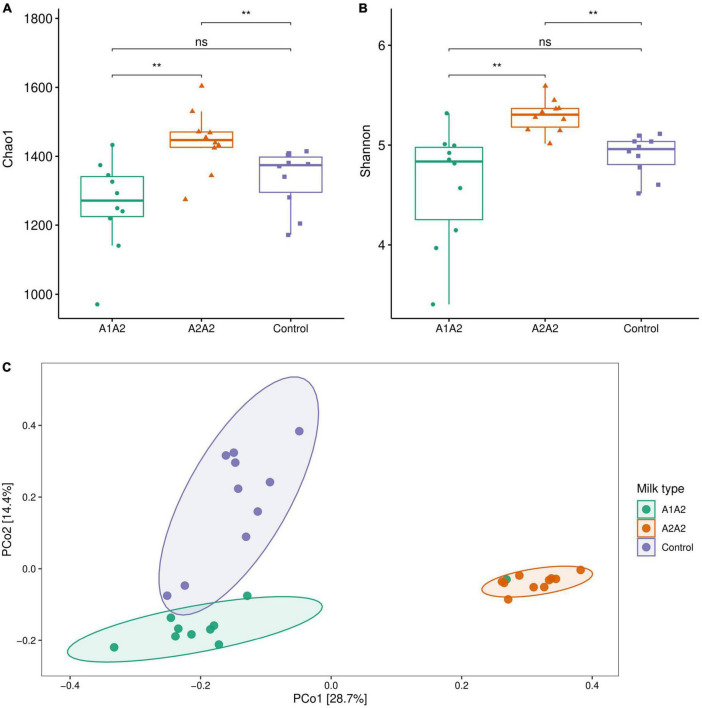
Alpha bacterial diversity metrics (**A**, Chao1; **B**, Shannon) and principle co-ordinate analysis (PCoA) of microbial genus level (Bray-curtis distance) **(C)** of mice on different types of β-casein milk administration. Control, standard chow feeding (without milk); A1A2, standard chow + bovine milk containing A1 and A2 β-casein; A2A2, standard chow + bovine milk containing only A2 β-casein. Each point represents a single sample and is colored by milk type. Each ellipse represents a group and is colored by milk type. **p < 0.01.

After 4-week intervention, A2A2 group showed significant increase of alpha-diversity indexes (Chao1 and Shannon) compared with A1A2 and control group (Figures 3A,B) (*p* < 0.05). PCoA showed that there was a clear separation of three groups, and A2A2 group clearly separated from A1A2 and control group, PC1 and PC2 together explained 43.1% of total difference (Figure 3C).

**FIGURE 4:**
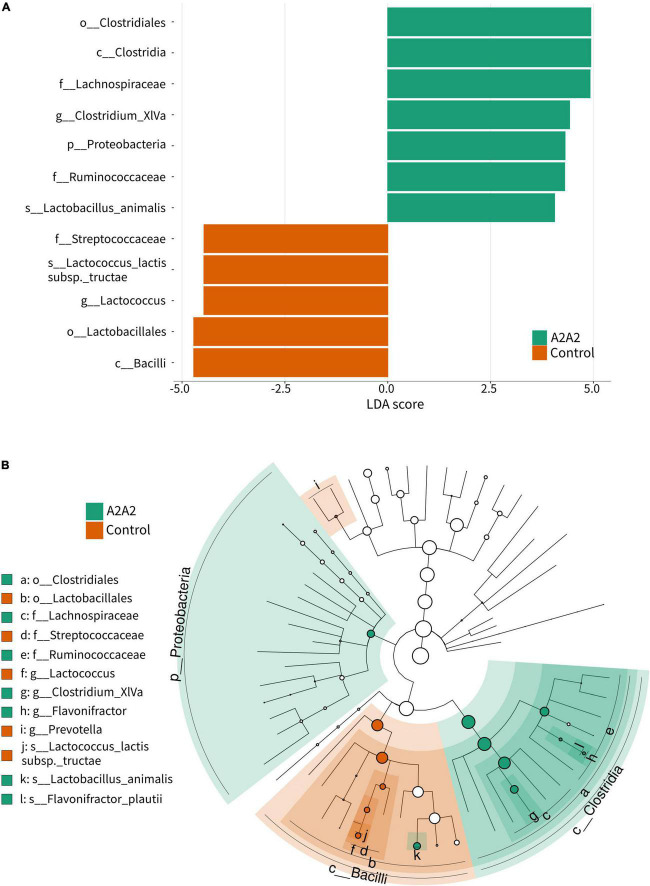
LEfSe analysis **(A)** and cladogram **(B)** of differential microbial genera of mice on different types of β-casein milk administration. Control, standard chow feeding (without milk); A1A2, standard chow + bovine milk containing A1 and A2 β-casein; A2A2, standard chow + bovine milk containing only A2 β-casein.

Differential analysis by LefSe showed that phylum Proteobacteria, class *Clostridia*, family *Ruminococcaceae*, and species *Lactobacillus animalis* were significantly higher in A2A2 group (*p* < 0.05), while class *Bacilli*, family *Streptococcaceae*, genus *Lactococcus* and species *Lactococcus lactis* subsp *tructae* were significantly higher in Control group (*p* < 0.05) (Figures 4A,B).

### Association analysis

**FIGURE 5:**
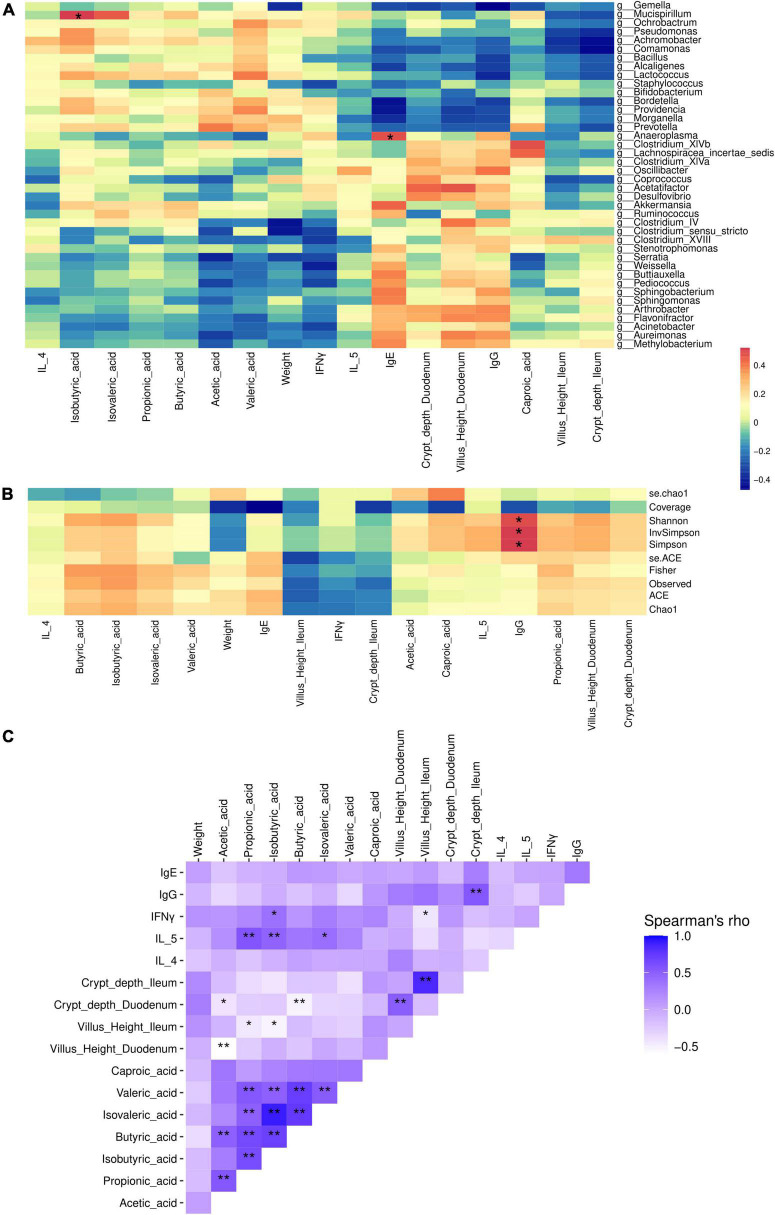
Association of microbial genera **(A)** and alpha-diversity **(B)**, with weight, intestinal morphology, immune factors, inflammation factors, and SCFAs **(C)** of mice on different types of β-case in milk administration. Control, standard chow feeding (without milk); A1A2, standard chow + bovine milk containing A1 and A2 β-casein; A2A2, standard chow + bovine milk containing only A2 β-casein. **p < 0.01, *p < 0.05.

To further confirm microbial effects on immune factors, SCFAs, and gut morphometric indices, Spearman correlation analysis were performed. The results showed that gut microbial genera *Anaeroplasma* was positively correlated with IgE (*p* < 0.05), and *Mucispirillam* was positively correlated with isobutyric acid (*p* < 0.05) (Figure 5A). IgG was positively correlated with microbial diversity (Shannon, InvSimpson, and Simpson index) (*p* < 0.05) (Figure 5B).

We also performed Spearman correlation between serum immune factors and inflammation factors, and gut SCFAs, and morphometric indices of small intestine. SCFAs showed significant medium correlation with IL-5 and IFN-γ (*p* < 0.05), and weak significant correlation with villus and crypt indices (*p* < 0.05). Also, IgG significantly correlated with crypt depth of ileum (*p* < 0.05) (Figure 5C).

## Discussion

Food proteins may provide biologically active peptides, which are important for host gastrointestinal functions. Our research characterized the effect of milk with variant β-casein types on immune, gut microbiota and intestinal morphology using mice model. We hypothesized that A2-type β-casein could provide gastrointestinal benefits and improve the immune and gut health.

We found that intake of A2-type β-casein milk significantly increase serum immune factors, such as IgE and IgG after 4 weeks of intervention, which means the effect of intake of A2-type milk could modulate the system immune responses. Whether A1-type or A2-type will cause allergic or inflammation still remains discussion. Some studies showed increased allergic or inflammation of A1-type milk, compared with A1A2 or A2A2 milk. IgE and IgG were significantly increased in serum of mice fed with A1-type milk (13), and also, compared with A2-type variants, consumption of A1-type β-casein induced gut inflammatory response by activating Th2 pathway; A1/A1 and A1/A2 milk administration showed significantly increased indicators of gut inflammation, such as IgE, IgG in interstinal fluid, compared with A2/A2 or control group (20); however, in another comprehensive study, serum IgG levels showed no differences among A1A1, A2A2, and control group (12). Moreover, compared with control, both milk showed no significant change of inflammation level, as IFN-γ, IL-4, and IL-5 showed no significant difference among three groups. Although some of the animal studies showed increased inflammatory effects of A1-type milk, different animal studies showed inconsistent results, this remains discussion and needs more future validation.

According to histological findings, A2-type β-casein milk intervention also showed increase of villus height and crypt depth of duodenum, compared with A1-type β-casein milk and control; but this effect was not found in ileum. This tissue-specific effect could be explained that milk casein are mainly digested in stomach and the proximal part of small intestine, so the digested peptides cannot reach the distal part of small intestine (21). A1-type β-casein in milk could be digested into BCM-7 peptide, and leads to delayed milk gastrointestinal transit time and health issue, which remains controversial. Also, Sanchón et al. showed that BCM-7 and other peptides were mainly found in jejunum after intake of β-casein (22). So the finding that A2-type casein consumption could improve intestinal morphology, and specifically in proximal small intestine.

A2-type β-casein milk could regulate gut microbiota and SCFAs. Guantario et al. have reported that A2-type β-casein milk altered gut microbial composition of aging mice, they showed that *Deferribacteriaceae* and *Desulfovibrionaceae* as the most discriminant families for the A2A2 group, while *Ruminococcaceae* were associated to the A1A2 group (12). We also found that consumption of different type of β-casein milk could affect microbiota and gut health in mice. Although microbiota profiles from A1- and A2-type β-casein milk treated mice and healthy controls were taxonomically similar, in-depth statistical analysis revealed that phylum Proteobacteria, class *Clostridia*, family *Ruminococcaceae*, and species *Lactobacillus animalis* were associated with A2-type β-casein milk, while class *Bacilli*, family *Streptococcaceae*, genus *Lactococcus* and species *Lactococcus lactis* subsp *tructae* were associated with healthy control. *Ruminococcaceae* is an important family of butyrate-producing bacteria. Despite of these microbial differences, their gut SCFAs showed no difference. Gut microbial SCFAs were reported to increase host antibody responses (23), and regulating the immune response and inflammation (24). Compared to A2 β-casein, A1 β-casein supplementation increased abundances of several diabetes-related bacterial species, such as *Streptococcus pyogenes* and *S. suis* in NOD mice (10). Although not significant in three groups, isobutyric acid was positively correlated with *Mucispirillam*. Moreover, *Anaeroplasma* was positively correlated with IgE, and IgG was positively correlated with Shannon, InvSimpson, and Simpson index. Previous study demonstrate that *Mucispirillum* is associated with a healthy gut (25), and the microbiota is correlated with intestinal barrier functions (26), nutrients metabolism, and maturation of host immune response (27). SCFAs showed significant medium correlation with IL-5 and IFN-γ, and weak significant correlation with villus and crypt indices. Also, IgG significantly correlated with crypt depth of ileum. IFN-γ is an cytokine secreted from CTLs, NK cells, NKT cells, and γδ T cells, which plays important role in immune response to infections (28). IL-5 is mostly generated by T helper-2 (Th2) lymphocytes and group 2 innate lymphoid cells (ILC2). It can increase antibody secretion and improve the humoral immune response by Th2 cells (29). This means gut microbes could potentially influence host immune responses, mediated by gut SCFAs, and may potentially led to immunological benefits.

### Limitations of the study

Gut microbiota of mice fecal was analyzed using 16S rRNA sequencing in this study, and future studies should use shotgun metagenomic sequencing to explore the effects of different types of β-casein in microbial genes and biological functions. Additionally, except for the fecal SCFAs, non-targeted metabolome profiling of fecal and serum should be implemented to illustrate the molecular mechanisms. The putative role of different types of β-casein in gastrointestinal comfort symptoms of mice may also be considered.

## Conclusion

In conclusion, diet supplementation of A2-type β-casein milk could change the composition and diversity of gut microbiota by increase the relative abundance of phylum Proteobacteria, class *Clostridia*, family *Ruminococcaceae*, and species *Lactobacillus animalis*, and improve proximal small intestine villus and crypt morphology. Associations of gut microbes-immune response-SCFA named isobutyric acid may potentially regulated those benefits. These findings illustrate potential specific benefits of consumption of A2-type β-casein milk on intestinal structure, and host immune system and gut health outcomes, which provide new aspect for nutritional intervention.

## Data availability statement

The data presented in this study are deposited in the Figshare repository, accession doi: 10.6084/m9.figshare.21076270.v1 (https://figshare.com/articles/dataset/A2CSN-MILK-MICE_zip/21076270).

## Ethics statement

Protocols were approved by the Animal Care and Use Committee of Sino Animal (Beijing) Science and Technology Development Co., Ltd., (20210077YZE-3R, Beijing, China). Experiment were performed following the Guide of Experimental Animals of the Ministry of Science and Technology (Beijing, China).

## Author contributions

LC: designed research. BL: performed statistical analysis and wrote manuscript. WQ and MZ: analyzed milk nutritional composition. YL: collected serum and fecal samples. JZ: conducted animal experiment. All authors have read and approved the final manuscript.
