# Supplementary material for: Bovine milk with variant β-casein types on immunological mediated intestinal changes and gut health of mice
Source: Front Nutr. 2022 Sep 30;9:970685. doi: 10.3389/fnut.2022.970685 (PMC9562473; doi:10.3389/fnut.2022.970685)
Supplement: Supplementary file 1 [file Table_1.DOCX]

**Additional Materials**

Additional File 1. Basic nutrients content (mean ± s.d.) in different types of milk.

| **Nutrients** | **A2A2 milk** | **A1A2 milk** |
| --- | --- | --- |
| β-casein *^1^* | 1.25 ± 0.05 | 1.20 ± 0.01 |
| A2 type β-casein *^1^* | 1.20 ± 0.06 | 0.76 ± 0.03 |
| ***Macronutrients ^1^*** |  |  |
| Protein | 3.32 ± 0.04 | 3.32 ± 0.10 |
| Fat | 3.89 ± 0.05 | 3.87 ± 0.03 |
| Lactose | 4.84 ± 0.10 | 4.92 ± 0.14 |
| ***Micronutrients*** |  |  |
| ***Amino acids ^1^*** |  |  |
| Alanine | 0.11 ± 0.01 | 0.11 ± 0.00 |
| Arginine | 0.11 ± 0.00 | 0.11 ± 0.01 |
| Aspartate | 0.25 ± 0.01 | 0.25 ± 0.01 |
| Cysteine | 0.02 ± 0.00 | 0.02 ± 0.00 |
| Glutamate | 0.65 ± 0.03 | 0.63 ± 0.03 |
| Glysine | 0.06 ± 0.01 | 0.06 ± 0.00 |
| Histine | 0.09 ± 0.00 | 0.09 ± 0.01 |
| Isoleucine | 0.18 ± 0.01 | 0.18 ± 0.00 |
| Leucine | 0.32 ± 0.01 | 0.32 ± 0.01 |
| Lysine | 0.27 ± 0.01 | 0.27 ± 0.01 |
| Methionine | 0.10 ± 0.00 | 0.10 ± 0.00 |
| Phenylaine alanine | 0.17 ± 0.01 | 0.17 ± 0.01 |
| Proline | 0.27 ± 0.06 | 0.27 ± 0.06 |
| Serine | 0.17 ± 0.01 | 0.16 ± 0.01 |
| Sucine | 0.15 ± 0.01 | 0.14 ± 0.01 |
| Tryptophan | 0.05 ± 0.01 | 0.05 ± 0.00 |
| Tyrosine | 0.16 ± 0.01 | 0.16 ± 0.01 |
| *Sum of 18 Amino acids* | 3.40 ± 0.13 | 3.37 ± 0.10 |
| ***Minerals ^2^*** |  |  |
| Calcium | 1032.00 ± 13.04 | 1082.00 ± 19.24 |
| Potassium | 1400.00 ± 18.71 | 1432.00 ± 36.33 |
| Magnesium | 101.60 ± 1.52 | 104.00 ± 2.00 |
| Natrium | 365.20 ± 3.83 | 376.60 ± 3.29 |
| Phosphorus | 852.40 ± 4.93 | 863.20 ± 19.46 |
| Zinc | 3.70 ± 0.05 | 4.07 ± 0.06 |

^1^ g/100 g; ^2^ mg/kg;

* All the nutrients (except A2 type beta-casein) showed no significant differences (T-test, pvalue < 0.05) between two types of milk.
